# Sweet Corn Stalk Treated with *Saccharomyces Cerevisiae* Alone or in Combination with *Lactobacillus Plantarum*: Nutritional Composition, Fermentation Traits and Aerobic Stability

**DOI:** 10.3390/ani9090598

**Published:** 2019-08-23

**Authors:** Xiaoling Zhou, Zhu Ouyang, Xiaoli Zhang, Yuqing Wei, Shaoxun Tang, Zhiyuan Ma, Zhiliang Tan, Nong Zhu, Tsegay Teklebrhan, Xuefeng Han

**Affiliations:** 1 CAS Key Laboratory for Agro-Ecological Processes in Subtropical Region, National Engineering Laboratory for Pollution Control and Waste Utilization in Livestock and Poultry Production, South Central Experimental Station of Animal Nutrition and Feed Science in the Ministry of Agriculture, Institute of Subtropical Agriculture, Chinese Academy of Sciences, Changsha 410125, China; 2 College of Advanced Agricultural Sciences, University of the Chinese Academy of Sciences, Beijing 100049, China; 3 Collge of Animal Science, Tarim University, Alaer 843300, China; 4 Key Laboratory of Ecosystem Network Observation and Modeling, Institute of Geographic Sciences and Natural Resources Research, Chinese Academy of Sciences, Beijing 100101, China; 5 Hunan Co-Innovation Center of Animal Production Safety, CICAPS, Changsha 410128, China

**Keywords:** *Saccharomyces cerevisiae*, *Lactobacillus plantarum*, Fermentation trait, Aerobic stability, Nutritional composition

## Abstract

**Simple Summary:**

The inclusion of *Saccharomyces* in the ration is beneficial to ruminants. We investigated the effects of inoculating a high-dose *S. cerevisiae* (10^8^ cfu/g) on the nutritional composition and fermentation traits of sweet corn stalk. A high-dose *S. cerevisiae* inoculum increased the crude protein concentration of sweet corn stalk silage but decreased the silage quality. Thus, a high-dose *S. cerevisiae* inoculum is not conducive to obtaining high-quality corn stalk silage.

**Abstract:**

This study examined the effects of a high-dose *Saccharomyces cerevisiae* inoculant alone or jointly with *Lactobacillus plantarum* on nutrient preservation, fermentation quality, and aerobic stability of sweet corn stalk silage. Fresh stalks (231 g dry matter (DM)/kg) were chopped and subjected to the following treatments: (1) deionized water (Uninoculated; U); (2) *S. cerevisiae* at 1 × 10^8^ cfu/g of fresh forage (S); and (3) *S. cerevisiae* at 1 × 10^8^ cfu/g plus *L. plantarum* at 1 × 10^5^ cfu/g (SL). Treated stalks were ensiled in 5-litre laboratory silos for 30, 60, and 90 day. The S and SL silages had a greater (*p* < 0.001) pH and greater crude protein, ammonia nitrogen/total nitrogen, neutral detergent fibre, acid detergent fibre, and ethanol contents at all three ensiling periods than the U silage. Acetate, propionate and volatile fatty acids in the S and SL silages after 30 and 90 day of ensiling were greater (*p* < 0.05) than those in the U silage, but they were lower (*p* < 0.05) in the S and SL silages than in the U silage after 60 day. The lactate and V-score of the S and SL silages were lower (*p* < 0.001) than those of the U silage at all three ensiling periods. Compared with the U group, the aerobic stability of the S silage after 90 day of ensiling decreased (*p* < 0.05), and the aerobic stability of the SL silage was unaffected (*p* > 0.05). Overall, the quality of sweet corn stalk silage was not improved by inoculation with 10^8^ cfu/g of *S. cerevisiae* alone or in combination with 1 × 10^5^ cfu/g of *L. plantarum*.

## 1. Introduction

The fresh sweet corn stalk, after harvesting the cob, is rich in protein, starch, and water-soluble carbohydrates [1], which are used as unconventional fodder in herbivores [2]. However, the nutritional value of corn stalk drops rapidly during withering or maturation [3]. Ensiling is an effective method to minimize the loss of nutrients during storage and preserve fresh forage for long-term use. The application of validated inoculants, especially lactic acid bacteria (LAB), reduces the risk of fermentation failure and prolongs shelf life [4,5]. The use of the first three generations of [6] inoculants (homofermentative LAB, heterofermentative LAB, and a combination of homo- and hetero-fermentative LAB) has mainly focused on improving the fermentation quality during sealing and the aerobic stability during the feedout phase [5]. In practice, the current demand for the next generation of silage inoculants is to further enhance the nutritional quality and to regulate intestinal microflora. *Saccharomyces cerevisiae* is a candidate microorganism with rich vitamins and growth factors [7]. It aids in modulating the immune system of young animals [8], improving rumen fermentation [9,10], and enhancing nutrient degradability of roughage in the hindgut [11]. However, *S. cerevisiae* cannot colonize the gastrointestinal tract [12], and it is intolerant of heat during feed processing [7,13]. Currently, viable *S. cerevisiae* is generally used in the form of active dry yeast powder and is mixed into the concentrate to feed [14,15,16], but the delivered viable count is variable [7,17]. The use of *S. cerevisiae* as a silage inoculant that can maintain or even enhance the number of viable cells is of great interest, as this will facilitate feeding management of *S. cerevisiae* and increase the feeding value of silage.

Some lactate-assimilating yeasts are considered to accelerate aerobic spoilage [5]. As a member of yeast, the idea of *S. cerevisiae* as a silage inoculant is still debated. Recently, two studies [18,19] found that when *S. cerevisiae* was inoculated into whole maize silage at a dose of 10^3^ to 10^5^ cfu/g of fresh forage, the population of *S. cerevisiae* in corn silage survived during ensiling and increased after feedout. The nutritional quality, fermentation traits, aerobic stability, and lactobacilli populations of corn silage were not influenced. Moreover, *S. cerevisiae* is not directly related to aerobic spoilage through microbial analyses [6,20]. These studies have shown the possibility of using *S. cerevisiae* as an inoculant at the dose of 10^3^ to 10^5^ cfu/g, but the application dose adopted in the literature and the resultant viable cell counts are below the dose suggested to elicit a positive response in the rumen [18,21]. The effect of an increased dose of *S. cerevisiae* on nutrient preservation, fermentation traits and aerobic stability remains unclear and needs to be assessed.

*S. cerevisiae* can secrete nitrogen-metabolic enzymes to effectively degrade exogenous amino acids and peptides into inorganic nitrogen, and inorganic ammonia plays a central role as an intermediate between degradative and biosynthetic pathways in nitrogen metabolism in *S. cerevisiae* [22]. Although the NH_3_–N and pH were not altered at the dose of 10^3^ to 10^5^ cfu/g of fresh forage [18,19], a short-term fermentation study showed that the inclusion of liquid brewer’s yeast at a high proportion raised the pH and ammonia nitrogen (NH_3_–N) in rice straw silage [23]. The concern that a high-dose inoculation with *S. cerevisiae* may increase plant protein degradation and impair nutritional quality of silage was raised. *Lactobacillus plantarum* is a facultative heterofermentative LAB, which mainly ferments hexoses to produce lactic acid and ferments pentoses to produce acetic acid, thereby reducing the pH and inhibiting ammonia-N production [5]. We hypothesized that inoculation with both *S. cerevisiae* and *L. plantarum* would rapidly lower the pH and favor the fermentation quality. Here, the effects of a high-dose *S. cerevisiae* inoculum alone or jointly with *L. plantarum* on the nutrient composition, aerobic stability, and fermentation traits of corn stalk silage for 30, 60, and 90 day were evaluated in this study.

## 2. Materials and Methods

### 2.1. Forage Harvest and Silage Preparation

Sweet corn (Nongda 108, Dabeinong Technology Group Co. Ltd., Beijing, China) was planted at Yucheng Research Station (116.57° E, 37.02° N), Shandong Province, China. After corncobs were harvested at the milky stage of maturity in August 2016, fresh corn stalks were mowed to a stubble height of 15–20 cm. The stalks were immediately chopped to an approximate length of 2 cm using a forage cutter (9Z-20, Wanying Machinery Equipment Co., Ltd., Zhengzhou, Henan Province, China). The chopped forage was treated with (1) deionized water (0.5 mL/kg of fresh forage) as the uninoculated control (U); (2) *S. cerevisiae* at 1 × 10^8^ cfu/g of fresh forage (Procreatin-7, Lesaffre, Marcq-en-Baroeul, France; S); and (3) *S. cerevisiae* at 1 × 10^8^ cfu/g and *L. plantarum* at 3 × 10^5^ cfu/g (Taiwan Yaxin Biotechnology Co. Ltd., Taiwan, China; SL). Procreatin-7 is a commercially available active dry yeast (15 × 10^9^ cfu/g of *S. cerevisiae*) powder. The application rate of *S. cerevisiae* was calculated according to the viable cells labelled in the instructions and sprinkled onto the forage with constant mixing by hand. Similarly, the theoretical inoculation amount of *L. plantarum* was diluted in sterile deionized water and applied at the rate of 0.5 mL/kg of fresh forage with a sprayer under constant manual mixing. Approximately 3.5 kg of treated forage was packed into 5-litre laboratory silos made of high-density polyethylene (19 cm in diameter × 27.5 cm in height) to achieve a packing density of 158 kg of dry matter (DM)/m^3^ and sealed airtight with a layer of polyethylene membrane and a thick screw top. Eighteen replicates of each treatment were then stored in an enclosed warehouse at ambient temperature (25 ± 1 °C) for 30, 60 and 90 day.

### 2.2. Sampling and pH Determination

After 30, 60, and 90 day of ensiling, six silos from each treatment were randomly opened. The content in each silo was mixed thoroughly and sampled for analysis. Silage extract was prepared by adding 25 g of corn stalk silage to 225 mL deionized water, storing the sample for 24 h at 4 °C and then manually homogenizing the sample for 2 min according to the method described by Wang et al. [24]. The pH of the water extract was measured using a pH meter (FiveEasy Benchtop pH Meter FE20, Mettler-Toledo, Columbus, OH, USA). Then, the solution was filtered through Whatman 54 filter paper, and the filtered water extract was subdivided into 10 mL aliquots. Two aliquots were used directly for ammonia and water-soluble carbohydrates (WSCs) analysis, and three aliquots were frozen at −20 °C after adding 2 mL of 0.3 mol/L metaphosphoric acid for ethanol and organic acid analyses.

### 2.3. Determination of Chemical Composition 

Approximately 300 g of silage samples in each silo were used in the determination of chemical composition, including neutral detergent fibre (NDF), acid detergent fibre (ADF), total nitrogen (TN), and starch. The DM of the silage samples was dried using a forced-air oven at 60 °C for 48 h and then ground with a pulveriser (FW-100, Beijing Yongguangmin Ltd., Beijing, China) prior to being passed through a 1-mm screen for further analysis. The DM concentration was corrected for the loss of volatile compounds according to Porter and Murray [25], and the variables after ensiling were presented on the basis of corrected DM. Crude protein (CP) was calculated by determining TN using a flow injection technology (AutoAnalyzer3, Flow Injection Analysis System, Seal Analytical, Norderstedt, Germany) according to the digestion procedure of the AOAC-2001.11 method (AOAC 2002) [26] and using a fixed conversion factor (6.25). Ether extract (EE) was also analysed using an automatic extraction apparatus (SOX416, C. Gerhardt GmbH & Co. KG, Königswinter, Germany) according to the AOAC 2003.05 method (AOAC 2006) [27]. The amounts of NDF and ADF were measured in a Fibretherm FT 12 Fiber Analyzer (C. Gerhardt GmbH & Co. KG) using the methods described by Mertens [28] with the inclusion of a heat-stable amylase and sodium sulphite. Both NDF and ADF were expressed inclusive of residual ash, and hemicellulose was calculated as the difference between NDF and ADF. Starch was analysed using the amyloglucosidase hydrolysis method as described by Wang et al. [29]. WSCs analysis based on the water extract of silage plant tissue homogenate was performed according to DuBois et al. [30].

### 2.4. Determination of Ammonia Nitrogen, Ethanol and Organic Acid Contents

After the frozen water extract was thawed, ammonia nitrogen (NH_3_–N) concentration was determined by the phenol-hypochlorite procedure as described by Chen et al. [31]. For the determination of ethanol and organic acid, the thawed frozen water extract was centrifuged at 15,000× *g* for 10 min at 4 °C, then the supernatant was filtered using a 0.22-μm syringe filter and transferred into a 2.0-mL glass chromatograph vial. Then, lactate was analysed by high-performance liquid chromatography (Agilent HPLC 1290, Agilent, Santa Clara, CA, USA) equipped with a Zorbax SB-C18 column (150 mm × 4.6 mm ID, 5 µm) and a diode array detector (G4212A, Agilent) and qualified by an external standard method with five standard solutions (0.01, 0.05, 0.10, 0.15 and 0.20 mg/mL). Mobile phase A was a 19.1 mmol/L H_3_PO_4_ solution, and mobile phase B was HPLC grade acetonitrile. Both mobile phases were applied at a flow rate of 1 mL/min. The column temperature was maintained at 25 °C, and the detection wavelength was 210 nm. The system calibration and integrity were evaluated through the periodic injection of standard lactate solutions. Ethanol and volatile fatty acids (VFAs, including acetate, propionate and butyrate) were analysed using a gas chromatograph (Agilent 7890A, Agilent Inc.) equipped with a flame ionization detector and a DB-FFAP column (30 m × 0.25 mm × 0.2 µm), and the detailed procedures were described by Playne [32].

### 2.5. Background Microbial Population Analysis

Approximately 5 g of frozen fresh forage was ground in liquid nitrogen, and 100 mg subsamples were used for DNA extraction using a commercial kit (QIAamp fast DNA stool mini kit; QIAGEN GmbH, Hilden, North Rhine-Westphalia, Germany) according to the manufacturer’s instructions. The concentration and purity of total DNA were measured using an ND-2000 Spectrophotometer (NanoDrop Technologies, Wilmington, DE, USA). The extracted DNA was diluted with ultrapure water to 1 ng/μL, and PCRs were performed in triplicate. Each reaction was performed in a 20 μL mixture containing 0.8 μL of each primer (10 µM), 10 ng of template DNA, 2 μL of 2.5 mM dNTPs, 0.4 μL of FastPfu polymerase (TransGen Biotech Co., Ltd., Beijing, China) and 4 μL of 5 × FastPfu Buffer (TransGen Biotech Co., Ltd.). Specific primers containing an Illumina adaptor and barcode sequences were designed for the 16S rDNA V3–V4 hypervariable regions (341F, 5’- CCTAYGGGRBGCASCAG-3’; 806R, 5’- GGACTACNNGGGTATCTAAT-3’) of the bacterial genomic DNA. The PCR products were excised from 2% agarose gels and purified using a QIAquick Gel extraction kit (QIAGEN GmbH). Amplicons from each reaction mixture were quantified fluorometrically, normalized and pooled at equimolar ratios based on the concentration of each amplicon. The amplicons were sequenced (paired-end) on an Illumina HiMiSeq PE300 platform by Novogene Company (Beijing, China). The 16S sequence reads were demultiplexed, the adaptors and barcodes were trimmed, and contigs were obtained based on the overlaps of pair-ended sequences using QIIME (v 1.7.0). The contigs were filtered according to quality using QIIME (v 1.7.0) as described by Caporaso et al. [33]. Chimeras were filtered using UCHIME v 11 against the Gold database [34]. The high-quality contigs were uniquified and clustered into operational taxonomic units (OTUs) at 97% similarity using USEARCH v.10 [35]. Representative sequences were defined based on the abundance of each OTU. A taxonomy analysis was performed using the SILVA database v.132 [36] with a minimum support threshold of 80%.

### 2.6. Aerobic Stability and Quality Assessment

After 90 day of ensiling, the silos were opened, and samples were collected. Two kg of silage was returned to the silo for the measurement of aerobic stability. Silages were covered with two layers of cheesecloth to prevent drying and contamination but to allow the penetration of air. Thermocouple probes (i500-E3TW, Yuhuanzhituo Instrumental Co., Ltd, Hangzhou, Zhejiang Province, China) were placed at the geometric centre of each sample to collect the temperature every 60 min; the ambient temperature was synchronously detected by another probe. Aerobic stability was denoted as the length of time that elapsed before the silage and ambient temperatures differed by more than 2 °C [37]. The V-score evaluation system [38] was applied to assess the quality score of the corn stalk silage based on the parameters of NH_3_–N and organic acids.

### 2.7. Data Analysis

Statistical analyses were performed using SPSS 19.0 statistical software (IBM Corp., Armonk, NY, USA, 2010). The linear mixed procedure was performed to examine the differences as follows: Y_ijk_ = μ + T_i_ + P_j_ + T_i_ × P_j_ + e_ijk_, where Y_ijk_ is the dependent variable, μ is the overall mean, T_i_ is the fixed effect of the treatment, P_j_ is the fixed effect of the ensiling period, T_i_ × P_j_ is the interaction between T_i_ and P_j_, and e_ijk_ is the random residual error. Aerobic stability data were analysed by the mixed model with the fixed effect of treatments. Differences among means were tested using the *Bonferonni* comparison [39], and significant differences were declared at *p* < 0.05. Data are presented as the means and standard error of the means (SEM).

A principal component analysis (PCA) was performed using SIMCA 14.0 software (Umetrics AB, Umeå, Sweden) based on the variables of the pH value, the chemical composition, organic acids, and ethanol. PCA-X and partial least squares-discriminant analysis (PLS-DA) were auto-fitted using seven-fold cross validations. The eigenvalue similarity level and significance level for DModX and Hotelling’s T2 were both at 0.05. The variable importance in the projection (VIP) score in the PLS-DA model was analysed, and the “VIP scores greater than one” rule was generally used as the criterion for important variable selection [40], that is, a higher VIP score indicates a greater importance of this variable for explaining the difference among groups.

## 3. Results

### 3.1. Chemical Composition and Microbial Population of Fresh Forage

The fresh stalk contained 247.1 g DM/kg as fed, 134.1 g/kg WSCs, 667.8 g/kg NDF, 426.3 g/kg ADF, 80.8 g/kg starch, 18.1 g/kg EE, and 72.3 g/kg CP on a DM basis. In the fresh stalk, the top five genera of the epiphytic microorganism population were *Pseudomonas*, *Pantoea*, *Klebsiella*, *Raoultella*, and *Enterobacter* (Table 1), which accounted for 73.18% of the total sequences. The majority of the population belonged to the phyla *Proteobacteria*, *Bacteroidetes Firmicutes* and *Actinobacteria*, which accounted for 86.96, 1.59, 0.81 and 0.29% of the total sequences, respectively. The epiphytic LAB included 0.35% *Lactobacillus*, 0.24% *Lactococcus*, 0.22% *Weissella* and a trace amount (0.018%) of *Enterococcus*.

### 3.2. Chemical Composition of Silage

The DM content in the SL group was lower (*p* < 0.05) than that in the U and S groups after 30 day of ensiling (Table 2), while it was similar between treatments after 30 and 60 day of ensiling. The concentrations of CP, NH_3_-N/TN, NDF and ADF in the S and SL groups during 90 day of ensiling increased (*p* < 0.001) compared with the U group, while the starch and WSCs concentrations in the S and SL groups decreased (*p* < 0.001) compared with those in the U group. Compared with the hemicellulose concentrations in the U group, the hemicellulose concentrations in the S and SL groups were unaffected (*p* > 0.05) at all three periods; only the concentration of hemicelluloses in the S group was lower than that in the SL group after 90 day of ensiling.

### 3.3. Fermentation Traits, Aerobic Stability and V-Score

The pH values in the S and SL silages were greater (*p* < 0.001) than those in the U group at all three ensiling periods (Table 3), and the pH values in the SL silages were highest (*p* < 0.001) at all three ensiling periods. The concentrations of acetate, propionate and VFAs in the S and SL groups were greater (*p* < 0.05) than those in the U group after 30 and 90 day of ensiling but lower (*p* < 0.05) in the S and SL groups than in the U group after 60 day of ensiling. The butyrate concentrations in the S and SL groups after 60 day of ensiling were lower (*p* < 0.05) than those in the U group, but they were higher in the SL group after 90 day of ensiling than in the U and S groups. However, the lactate concentration and lactate:acetate ratio in the S and SL groups after 30 and 90 day of ensiling were lower (*p* < 0.001) than those in the U group, while those values in the S and SL groups after 60 day of ensiling were higher (*p* < 0.001) than those in the U group. The ethanol concentrations in the S and SL silages were nine- to 29-fold greater (*p* < 0.001) than those of the U group at all three ensiling periods. The V-scores of the S and SL groups at all three ensiling periods were lower (*p* < 0.001) than those in the U group. Aerobic stability in the S group after 90 day of ensiling was lower (*p* < 0.05) than that in the U and SL groups.

### 3.4. Principal Component Analysis and VIP Score

**Figure 1:**
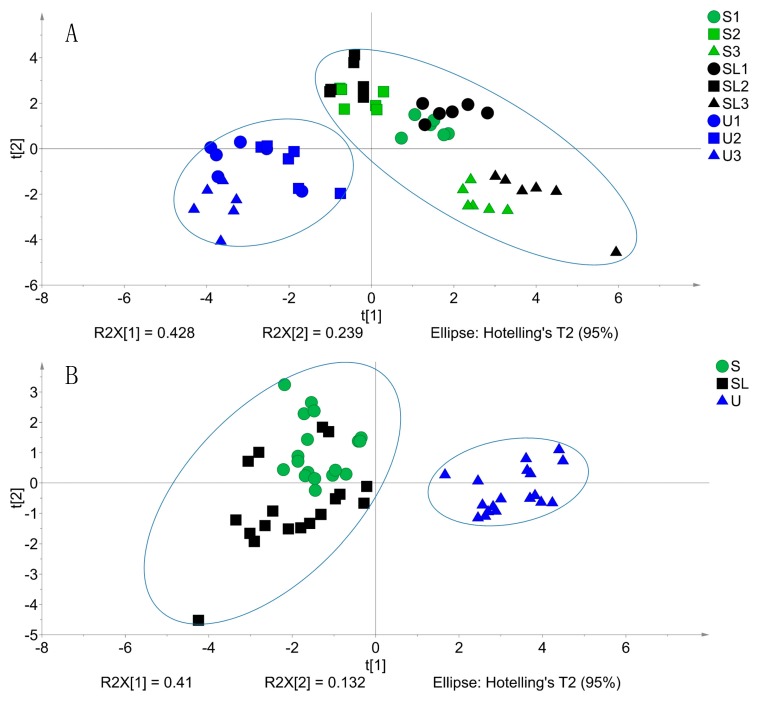
Scatter plots of the principal component analysis of the corn stalk silages inoculated with *Saccharomyces cerevisiae* alone or jointly with *Lactobacillus plantarum*. (**A**) PCA-X model; (**B**) PLS-DA model. U1/2/3 = no inoculant after 30, 60, or 90 day of ensiling, respectively; S1/2/3 = *S. cerevisiae* at 1 × 10^8^ cfu/g after 30, 60, or 90 day of ensiling, respectively; SL1/2/3 = *S. cerevisiae* at 1 × 10^8^ cfu/g and *L. plantarum* at 1 × 10^5^ cfu/g after 30, 60, or 90 day of ensiling, respectively. t[1]/[2] = Principal component 1 or 2.

**Figure 2:**
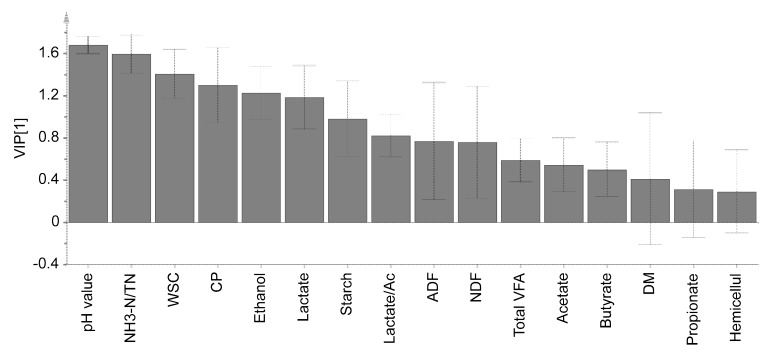
Histogram plot of the variable importance for the projection (VIP) in corn stalk silage inoculated with *Saccharomyces cerevisiae* alone or jointly with *Lactobacillus plantarum*. VIP [1] = VIP score based on principal component 1. Confidence intervals for the VIP values at the 95% level are shown as vertical bars. NH_3_-N/TN: Percentage of ammonia nitrogen to total nitrogen. WSCs = Water-soluble carbohydrates. Hemicellul: Hemicelluloses. VFAs include acetate, propionate, and butyrate. Lactate/Ac = Lactate:acetate ratio.

PCA-X analysis indicated the difference between the uninoculated and inoculated groups (Figure 1A, cumulative R^2^X = 0.798, cumulative Q^2^ = 0.615), but no clear separation among the three ensiling periods was found. This separation was further verified by the PLS-DA model (Figure 1B, cumulative R^2^Y = 0.705, cumulative Q^2^ = 0.654) after a valid permutation test (Q^2^ intercepts = −0.335), which showed the difference between the uninoculated and inoculated groups. According to the VIP score (>1) shown in Figure 2, the order of importance in fermentation variables was listed as follows: pH > NH_3_-N/TN > WSCs > CP > ethanol > lactate. The pH and NH_3_-N/TN (VIP score > 1.5) were the most important variables for explaining the differences among treatments.

## 4. Discussions

The present study indicated that the *S. cerevisiae* inoculant at a rate of 10^8^ cfu/g alone or together with *L. plantarum* affected the pH, chemical composition, organic acid profile, and ethanol content of corn stalk silage and led to a lower V-score. In contrast to our hypothesis, the combined inoculation of high-dose *S. cerevisiae* and *L. plantarum* further raised the pH and NH_3_-N levels, which did not facilitate the storage of fresh stalk.

The chemical composition of fresh sweet corn stalk in this study was typical of low DM (<25 g/kg) and high WSCs (>130 g/kg), but the level of WSCs was slightly lower than that of another breed of sweet corn stalk (171 g/kg) [41]. The NDF, ADF and CP concentrations were similar to those of corn stover in previous studies [42]. The background microbial population detected in this study mostly comprised the phyla *Proteobacteria*, *Firmicutes* and *Bacteroidetes*, which is consistent with the background microflora in 10% bloom alfalfa [43] and in corn stover [44]. *Lactobacillus*, *Lactococcus*, *Weissella*, and *Enterococcus* were the only four LAB detected in this study and accounted for 0.83% of the total sequences, which was much lower than the relative abundance of background *Lactobacillaceae* (2.43%) from 16S rDNA sequence data reported by Romero et al. [45].

PCA analysis is an effective tool to identify discriminative metabolites or markers after experimental intervention and has recently been used to assess silage quality [46]. Although the VIP score of the detected variables did not provide any additional biological significance, it deepened our understanding of the experimental results and demonstrated that the pH and NH_3_-N/TN among the detected variables were the most influential parameters to differentiate whether the silage had been inoculated with *S. cerevisiae*. The NH_3_-N/TN concentration reflects the extent of organic nitrogen degradation or proteolysis during silage [47], which is usually lower than 10% for gramineous crop silage [48]. The NH_3_-N/TN concentration of the uninoculated corn stalk silage in this study was comparable to the data reported by Li et al. [42] for corn stover silage. However, the NH_3_-N/TN concentration in the inoculated silages in this study was increased by two-fold compared with the uninoculated silage. Nitrogen sources, including inorganic nitrogen and free amino acids, are degraded by *S. cerevisiae* to yield ammonia as a precursor nitrogen source for protein anabolism [22], which may explain the higher concentration of NH_3_-N/TN in the silages containing *S. cerevisiae* in the present study. Generally, the conversion between inorganic nitrogen and organic nitrogen in silage does not influence TN and thus, does not influence the CP concentration because CP is estimated from the TN concentration. However, the CP sources in the silages inoculated with *S. cerevisiae* differed from the uninoculated silage in that the protein was derived not only from plant proteins but also from the microbial proteins of the inoculants, especially from *S. cerevisiae* cells with a protein content between 40% and 60% [49]. Hence, the high application size of *S. cerevisiae* (32.5 g/kg DM) was estimated to provide silage with an additional 13 to 19 g/kg DM of microbial protein, which almost explained the increase in CP in the inoculated silages. Similarly, Kamphayae et al. [23] reported that the CP content of the fermented end-product increased in proportion to the inclusion of increased brewer’s yeast during the short ensiling of a total mixed ration containing cassava pulp. However, it is not clear whether the increase in the CP concentration was accompanied by the proliferation of the *S. cerevisiae* population and the increase in true microbial protein.

Inoculation with *S. cerevisiae* alone or in combination with *L. plantarum* had little effect on the starch content, although significant differences occurred between the uninoculated silage and the silage inoculated with *S. cerevisiae* alone. This was due to the slower rate of degradation of the starch caused by the limited amount of amylase naturally secreted by *S. cerevisiae* [50]. However, *S. cerevisiae* and LAB, including *L. plantarum*, can effectively consume WSCs [51]; thus, the WSCs in the silages inoculated with high-dose *S. cerevisiae* alone or combined with *L. plantarum* in the present study were reduced. Structural carbohydrates, including hemicellulose, cellulose, and lignin, cannot be directly utilized by *S. cerevisiae* unless they are effectively saccharified [52]. The increase in NDF and ADF in the silages inoculated with *S. cerevisiae* could be largely due to the relative decrease in fermentable carbohydrates, including WSCs and starch, and DM loss. Unfortunately, we did not determine DM loss.

When forage with high moisture is ensiled, it is difficult to lower the pH value below the critical threshold of 3.8–4.2 [20,47] and inhibit the growth of undesired microorganisms such as butyric acid-producing *Clostridium*. Although the moisture content of fresh forage was up to 75% in this study, the pH value was below 3.8 in the control silages during 90 day of ensiling, which is indicative of a favourable ensiling process. However, the pH values of silages inoculated with *S. cerevisiae* alone or jointly with *L. plantarum* ranged from 4.3 to 4.6 during 90 day of ensiling. Many studies have confirmed that the pH value in silage is primarily dependent on lactic acid concentration and is partially affected by the VFA concentration. However, the concentrations of lactate and VFAs in the inoculated silages were inadequate in explaining the high pH. We postulated that the pH in the inoculated silages was mainly affected by the high levels of NH_3_–N/TN. Because ammonia is easily dissolved in water, the ammonia solution is weakly alkaline (the pKb for ammonia is 4.74), which counteracts the role of lactate in reducing the pH in the inoculated silages. However, Duniere et al. [18] reported no effect on pH value and NH_3_–N when *S. cerevisiae* was applied at a dose of 10^3^ cfu/g of fresh forage. This inconsistency is possibly due to the difference in application dose of *S. cerevisiae*. Additional supportive evidence is provided by Kamphayae et al. [23], who observed an increase in pH when the proportion of liquid brewer’s yeast included in the mixed silage of cassava pulp and rice straw increased. Thus, for a high inoculation dose of *S. cerevisiae*, a method by which the ammonia production can be reduced in silage must first be identified.

Previously, inoculation with *S. cerevisiae* had no impact on the fermentation products (acetate, propionate, lactate, succinic acid, ethanol, etc.) of corn silage at the application rate of 10^3^ to 10^5^ cfu/g [18,19], but inoculation with *S. cerevisiae* at the dose of 10^8^ cfu/g in corn stalk silages affected the ethanol and organic acid contents in the present study. High concentrations of ethanol in silages (>3–4%) are often associated with high numbers of yeasts [48], and the increase in the ethanol levels of the inoculated silages in this study reflected the active anaerobic metabolism of *S. cerevisiae*, suggesting that the survival or even proliferation of *S. cerevisiae* occurred during ensiling in this study, which was confirmed by 16S rDNA sequencing analysis in previous studies [18,19]. A high concentration of ethanol could lower the palatability of silage and affect milk flavour [48]; however, this weakness is not an issue if the ensiling of sweet corn stalk is used as a pre-treatment process for biomass ethanol, and this notion was tested with *L. plantarum* A1 with ferulic acid esterase activity in dry corn stover [42]. In addition, the acetate and propionate concentrations in the inoculant silages after 30 and 90 day of ensilage increased, which may be attributed to the high pH that cannot inhibit the metabolism of facultative aerobic bacteria, such as epiphytic *Pantoea, Klebsiella*, and *Enterobacter*. However, the reason for the abrupt decline in acetate and propionate in inoculated silages on 60 day of ensiling remains unclear and needs to be further verified. In contrast to the uninoculated silage, lactate was reduced in the silages inoculated with *S. cerevisiae*. Despite the fact that *S. cerevisiae* cannot utilize lactate [20], *S. cerevisiae* stimulates the growth of lactate-utilizing bacteria [51], which may partly explain the decrease in lactate. On the other hand, *S. cerevisiae* competes for the utilization of WSC substrates with LAB, which may limit lactate production by LAB. Notably, compared with the uninoculated silage, *S. cerevisiae* inoculant had no obvious effect on butyrate production during the first 60 day of ensiling; the butyrate increased only in the silage inoculated with the combined inoculants of *S. cerevisiae* and *L. plantarum* after 90 day of storage, which partly impaired the fermentation quality.

Aerobic stability is one of the key traits of silage preparation. After opening the silos, the elapsed time for aerobic deterioration depends on the initial microbial compositions [41], lactate concentration and pH [53,54,55], and higher amounts of VFAs (acetate, propionate, and butyrate) and NH_3_–N inhibit the growth of aerobic bacteria, yeasts, and moulds [48]. The reduced aerobic stability of the silage inoculated with *S. cerevisiae* alone was mainly due to the high pH value (4.3–4.4) that could not inhibit the growth of aerobic spoilage bacteria after opening. This result is consistent with the outcome of a high pH and poor stability observed by Kamphayae et al. [23] when a high proportion of liquid brewer’s yeast was included. In contrast, a lower inoculation dose (10^3^–10^5^ cfu/g) of *S. cerevisiae* did not affect the pH and aerobic stability of corn silage [18,19]. Therefore, poor aerobic stability is directly related to the high pH associated with NH_3_–N levels. However, aerobic stability was not influenced by the joint inoculation of *S. cerevisiae* and *L. plantarum* compared with the control in our study. The greater levels of propionate and butyrate may prolong the aerobic stability because propionate and butyrate have been accepted to have strong antifungal characteristics [48].

## 5. Conclusions

This research provides a new exploration of high-dose *S. cerevisiae* as an inoculant to ensile fresh forage. At 10^8^ cfu/g, although the *S. cerevisiae* inoculant increased the CP concentration of the corn stalk silage, it did not improve the quality of the corn stalk silage, as the silage had a high pH and a high concentration of NH_3_–N/TN, irrespective of *L. plantarum*. Further studies should optimize the inoculum size of *S. cerevisiae* in silage and preventative measures to inhibit ammonia production.

## Figures and Tables

**Table 1:** Relative abundance of the top 25 bacterial genera identified in fresh forage by 16S rDNA sequencing.

Genus	Relative Abundance (%)
*Pseudomonas*	46.70
*Pantoea*	14.37
Unclassified	8.02
*Klebsiella*	4.73
*Raoultella*	4.22
*Enterobacter*	3.15
*Stenotrophomonas*	2.59
*Sphingomonas*	2.19
*Acinetobacter*	1.79
*Sphingobacterium*	1.59
*Burkholderia*	1.50
*Delftia*	0.87
*Pectobacterium*	0.79
*Asaia*	0.73
*Serratia*	0.56
*Rhizobium*	0.55
*Ochrobactrum*	0.46
*Methylobacterium*	0.44
*Gluconobacter*	0.43
*Tatumella*	0.38
*Lactobacillus*	0.35
*Curtobacterium*	0.29
*Herbaspirillum*	0.25
*Lactococcus*	0.24
*Brevundimonas*	0.24
Total	97.45

**Table 2:** Chemical composition of the corn stalk silage ensiled for 30, 60, and 90 day (DM basis).

Item ^2^ (g/kg)	Days	Treatment ^1^			SEM ^3^	*p*-Value		
		U	S	SL		Treatment	Period	Interaction
DM	30	269 ^Aa^	260 ^Aa^	244 ^Bb^	2.7	<0.001	<0.001	0.048
	60	263 ^a^	256 ^a^	256 ^a^				
	90	223 ^b^	219 ^b^	213 ^c^				
CP	30	65 ^B^	85 ^Ab^	80 ^Aa^	1.2	<0.001	<0.001	<0.001
	60	65 ^C^	80 ^Ac^	72 ^Bb^				
	90	67 ^C^	93 ^Aa^	84 ^Ba^				
NH_3_-N/TN	30	55.8 ^C^	138.6 ^Bb^	177.2 ^Ab^	5.08	<0.001	<0.001	0.001
	60	63.8 ^B^	171.0 ^Aa^	177.4 ^Ab^				
	90	73.3 ^C^	177.7 ^Ba^	209.9 ^Aa^				
Starch	30	74.1 ^Aab^	72.6 ^Ba^	73.6 ^Aa^	0.18	<0.001	<0.001	0.021
	60	74.8 ^Aa^	72.8 ^Ba^	73.1 ^Ba^				
	90	72.9 ^Ab^	71.6 ^Bb^	72.1 ^ABb^				
WSCs	30	14 ^Ac^	4 ^B^	2 ^Bb^	0.9	<0.001	<0.001	<0.001
	60	21 ^Ab^	7 ^B^	8 ^Ba^				
	90	32 ^Aa^	6 ^B^	9 ^Ba^				
NDF	30	559 ^Ca^	592 ^Ba^	626 ^Aa^	7.8	<0.001	<0.001	0.072
	60	571 ^Ba^	610 ^Aa^	629 ^Aa^				
	90	451 ^Bb^	481 ^Bb^	548 ^Ab^				
ADF	30	373 ^a^	430 ^a^	412 ^a^	10.2	<0.001	<0.001	0.101
	60	389 ^Ba^	424 ^Aa^	429 ^Aa^				
	90	288 ^Cb^	328 ^Bb^	363 ^Ab^				
Hemicelluloses	30	187	161 ^ab^	214	9.2	0.001	0.003	0.450
	60	182	186 ^a^	200				
	90	163 ^AB^	153 ^Bb^	185 ^A^				

^A–C^ Means within the same row followed by different lowercase superscript letters are significantly different (*p* < 0.05); ^a–c^ Means within the same column of treatment or ensiling period followed by different lowercase superscript letters are significantly different (*p* < 0.05). ^1^ U, no inoculant; S, *Saccharomyces cerevisiae* at 1 × 10^8^ cfu/g; SL, *S. cerevisiae* at 1 × 10^8^ cfu/g and *Lactobacillus plantarum* at 1 × 10^5^ cfu/g. ^2^ DM, dry matter; CP, crude protein; NH_3_-N, ammonia nitrogen; TN, total nitrogen; WSCs, water-soluble carbohydrates; NDF, neutral detergent fibre; ADF, acid detergent fibre. ^3^ SEM, standard error of means.

**Table 3:** The pH, organic acids, V-score, and aerobic stability of corn stalk silages ensiled for 30, 60, and 90 day (DM basis).

Item	Days	Treatment ^1^			SEM ^2^	*p*-Value		
		U	S	SL		Treatment	Period	Interaction
pH	30	3.57 ^C^	4.42 ^B^	4.56 ^A^	0.021	<0.001	0.042	0.003
	60	3.66 ^C^	4.45 ^B^	4.57 ^A^				
	90	3.70 ^C^	4.37 ^B^	4.58 ^A^				
Acetate [g/kg]	30	10.5 ^Bb^	18.3 ^Ab^	21.1 ^Ab^	1.36	<0.001	<0.001	<0.001
	60	19.6 ^Aa^	8.7 ^Bc^	7.2 ^Bc^				
	90	8.6 ^Bb^	29.6 ^Aa^	28.8 ^Aa^				
Propionate [g/kg]	30	1.1 ^Ba^	1.7 ^Aa^	1.8 ^Aa^	0.12	0.028	<0.001	<0.001
	60	1.0 ^Aa^	0.3 ^Bc^	0.3 ^Bb^				
	90	0.4 ^Cb^	0.9 ^Bb^	1.5 ^Aa^				
Butyrate [g/kg]	30	0.5	0.7 ^a^	0.7 ^b^	0.26	<0.001	<0.001	<0.001
	60	0.5 ^A^	0.2 ^Bb^	0.2 ^Bc^				
	90	0.2 ^B^	0.4 ^Bab^	10.6 ^Aa^				
VFAs [g/kg]^3^	30	12.1 ^Bb^	20.6 ^Ab^	23.6 ^Ab^	1.59	<0.001	<0.001	<0.001
	60	21.2 ^Aa^	9.2 ^Bc^	7.7 ^Bc^				
	90	9.2 ^Bb^	30.9 ^Aa^	40.9 ^Aa^				
Lactate [g/kg]	30	64.2 ^A^	43.0 ^Bb^	44.5 ^Bab^	2.57	<0.001	0.001	0.222
	60	56.8 ^A^	44.4 ^Bb^	41.0 ^Bb^				
	90	63.9 ^A^	55.9 ^ABa^	50.7 ^Ba^				
Lactate:acetate ratio	30	5.6 ^Aa^	2.1 ^Bb^	1.9 ^Bb^	0.08	<0.001	0.014	<0.001
	60	2.7 ^Bb^	4.7 ^Aa^	5.3 ^Aa^				
	90	6.9 ^Aa^	1.8 ^Ba^	1.7 ^Bb^				
Ethanol [g/kg]	30	0.9 ^B^	16.5 ^Ab^	12.0 ^A^	3.00	<0.001	0.019	0.269
	60	1.8 ^C^	24.7 ^Aa^	16.6 ^B^				
	90	1.2 ^B^	34.8 ^Aa^	24.7 ^A^				
V-score	30	91.5 ^A^	61.5 ^Ba^	45.7 ^Ca^	2.22	<0.001	<0.001	<0.001
	60	90.3 ^A^	52.7 ^Bb^	49.8 ^Ca^				
	90	93.0 ^A^	46.5 ^Bb^	30.7 ^Bb^				
Aerobic stability [h]	90	106.8 ^A^	53.4 ^B^	124.8 ^A^	11.48	0.011	-	-

^A–C^ Means within the same row followed by different lowercase superscript letters are significantly different (*p* < 0.05); ^a–c^ Means within the same column followed by different uppercase superscript letters are significantly different (*p* < 0.05); ^1^ U, no inoculant; S, *Saccharomyces cerevisiae* at 1 × 10^8^ cfu/g; SL, *S. cerevisiae* at 1 × 10^8^ cfu/g and *Lactobacillus plantarum* at 1 × 10^5^ cfu/g. ^2^ SEM, standard error of means. ^3^ VFAs include acetate, propionate and butyrate.

## References

[1] Cui W., Dong Z., Zhang J., Wei J., Lin L., Zhang M. (2011). The nutrient components and ensilage fermentation quality of sweet corn stalks harvested at different times. ACTA Pratacult. Sin..

[2] Idikut L., Arikan B.A., Kaplan M., Guven I., Atalay A.I., Kamalak A. (2009). Potential nutritive value of sweet corn as a silage crop with or without corn ear. J. Anim. Vet. Adv..

[3] Zhang M., Wang Y., Tan Z., Li Z., Li Y., Lv H., Zhang B., Jin Q. (2017). Microorganism profile, fermentation quality and rumen digestibility in vitro of maize-stalk silages produced at different maturity stages. Crop Pasture Sci..

[4] Queiroz O.C.M., Ogunade I.M., Weinberg Z., Adesogan A.T. (2018). Silage review: Foodborne pathogens in silage and their mitigation by silage additives. J. Dairy Sci..

[5] Muck R.E., Nadeau E.M.G., McAllister T.A., Contreras-Govea F.E., Santos M.C., Kung L. (2018). Silage review: Recent advances and future uses of silage additives. J. Dairy Sci..

[6] Middelhoven W.J., van Baalen A.H.M. (1988). Development of the yeast flora of whole-crop maize during ensiling and during subsequent aerobiosis. J. Sci. Food Agric..

[7] Shurson G.C. (2018). Yeast and yeast derivatives in feed additives and ingredients: Sources, characteristics, animal responses, and quantification methods. Anim. Feed Sci. Technol..

[8] Trckova M., Faldyna M., Alexa P., Zajacova Z.S., Gopfert E., Kumprechtova D., Auclair E., D’Inca R. (2014). The effects of live yeast Saccharomyces cerevisiae on postweaning diarrhea, immune response, and growth performance in weaned piglets. J. Anim. Sci..

[9] Tang S.X., Tayo G.O., Tan Z.L., Sun Z.H., Shen L.X., Zhou C.S., Xiao W.J., Ren G.P., Han X.F., Shen S.B. (2008). Effects of yeast culture and fibrolytic enzyme supplementation on in vitro fermentation characteristics of low-quality cereal straws. J. Anim. Sci..

[10] Zhu W., Wei Z., Xu N., Yang F., Yoon I., Chung Y., Liu J., Wang J. (2017). Effects of Saccharomyces cerevisiae fermentation products on performance and rumen fermentation and microbiota in dairy cows fed a diet containing low quality forage. J. Anim. Sci. Biotechnol..

[11] Elghandour M.M.Y., Mellado M., Kholif A.E., Salem A.Z.M., Barbabosa A., Ballinas S., Esquivel A., Odongo N.E. (2016). Fecal gas production of ten common horse feeds supplemented with Saccharomyces cerevisiae. J. Equine Vet. Sci..

[12] Cordonnier C., Thévenot J., Etienne-Mesmin L., Denis S., Alric M., Livrelli V., Blanquet-Diot S. (2015). Dynamic In vitro models of the human gastrointestinal tract as relevant tools to assess the survival of probiotic strains and their interactions with gut microbiota. Microorganisms.

[13] Kwolek-Mirek M., Zadrag-Tecza R. (2014). Comparison of methods used for assessing the viability and vitality of yeast cells. FEMS Yeast Res..

[14] Ferraretto L.F., Shaver R.D., Bertics S.J. (2012). Effect of dietary supplementation with live-cell yeast at two dosages on lactation performance, ruminal fermentation, and total-tract nutrient digestibility in dairy cows. J. Dairy Sci..

[15] Muñoz C., Wills D.A., Yan T. (2017). Effects of dietary active dried yeast (Saccharomyces cerevisiae) supply at two levels of concentrate on energy and nitrogen utilisation and methane emissions of lactating dairy cows. Anim. Prod. Sci..

[16] Goncalves B.L., Goncalves J.L., Rosim R.E., Cappato L.P., Cruz A.G., Oliveira C.A.F., Corassin C.H. (2017). Effects of different sources of Saccharomyces cerevisiae biomass on milk production, composition, and aflatoxin M-1 excretion in milk from dairy cows fed aflatoxin B-1. J. Dairy Sci..

[17] Sullivan M.L., Bradford B.J. (2011). Viable cell yield from active dry yeast products and effects of storage temperature and diluent on yeast cell viability. J. Dairy Sci..

[18] Duniere L., Jin L., Smiley B., Qi M., Rutherford W., Wang Y., McAllister T. (2015). Impact of adding Saccharomyces strains on fermentation, aerobic stability, nutritive value, and select lactobacilli populations in corn silage. J. Anim. Sci..

[19] Xu S., Yang J., Qi M., Smiley B., Rutherford W., Wang Y., McAllister T.A. (2019). Impact of Saccharomyces cerevisiae and Lactobacillus buchneri on microbial communities during ensiling and aerobic spoilage of corn silage1. J. Anim. Sci..

[20] Santos M.C., Golt C., Joerger R.D., Mechor G.D., Mourao G.B., Kung L. (2017). Identification of the major yeasts isolated from high moisture corn and corn silages in the United States using genetic and biochemical methods. J. Dairy Sci..

[21] Kumprechtova D., Illek J., Julien C., Homolka P., Jancik F., Auclair E. (2019). Effect of live yeast (Saccharomyces cerevisiae) supplementation on rumen fermentation and metabolic profile of dairy cows in early lactation. J. Anim. Physiol. Anim. Nutr. (Berl).

[22] ter Schure E.G., van Riel N.A., Verrips C.T. (2000). The role of ammonia metabolism in nitrogen catabolite repression in Saccharomyces cerevisiae. FEMS Microbiol. Rev..

[23] Kamphayae S., Kumagai H., Bureenok S., Narmseelee R., Butcha P. (2017). Effects of graded levels of liquid brewer’s yeast on chemical composition and fermentation quality in cassava pulp and rice straw-based total mixed ration silage. Anim. Sci. J..

[24] Wang S., Li J., Dong Z., Chen L., Yuan X., Shao T. (2018). The effects of lactic acid bacteria strains isolated from various substrates on the fermentation quality of common vetch (*Vicia sativa* L.) in Tibet. Grass Forage Sci..

[25] Porter M.G., Murray R.S. (2001). The volatility of components of grass silage on oven drying and the inter-relationship between dry-matter content estimated by different analytical methods. Grass Forage Sci..

[26] Association of Official Analytical Chemists (2002). Official Methods of Analysis of AOAC International.

[27] Association of Official Analytical Chemists (2005). Official Methods of Analysis of AOAC International.

[28] Mertens D.R. (2002). Gravimetric determination of amylase-treated neutral detergent fiber in feeds with refluxing in beakers or crucibles: collaborative study. J. AOAC Int..

[29] Wang M., Wang R., Xie T.Y., Janssen P.H., Sun X.Z., Beauchemin K.A., Tan Z.L., Gao M. (2016). Shifts in rumen fermentation and microbiota are associated with dissolved ruminal hydrogen concentrations in lactating dairy cows fed different types of carbohydrates. J. Nutr..

[30] DuBois M., Gilles K.A., Hamilton J.K., Rebers P.A., Smith F. (1956). Colorimetric method for determination of sugars and related substances. Anal. Chem..

[31] Chen L., Ren A., Zhou C., Tan Z. (2016). Effects of Lactobacillus acidophilus supplementation for improving in vitro rumen fermentation characteristics of cereal straws. Ital. J. Anim. Sci..

[32] Playne M.J. (1985). Determination of ethanol, volatile fatty acids, lactic and succinic acids in fermentation liquids by gas chromatography. J. Sci. Food Agric..

[33] Caporaso J.G., Kuczynski J., Stombaugh J., Bittinger K., Bushman F.D., Costello E.K., Fierer N., Peña A.G., Goodrich J.K., Gordon J.I. (2010). QIIME allows analysis of high-throughput community sequencing data. Nat. Methods.

[34] Edgar R.C., Haas B.J., Clemente J.C., Quince C., Knight R. (2011). UCHIME improves sensitivity and speed of chimera detection. Bioinformatics.

[35] Edgar R.C. (2010). Search and clustering orders of magnitude faster than BLAST. Bioinformatics.

[36] Yilmaz P., Parfrey L.W., Yarza P., Gerken J., Pruesse E., Quast C., Schweer T., Peplies J., Ludwig W., Glockner F.O. (2014). The SILVA and “All-species Living Tree Project (LTP)” taxonomic frameworks. Nucleic Acids Res..

[37] Queiroz O.C.M., Arriola K.G., Daniel J.L.P., Adesogan A.T. (2013). Effects of 8 chemical and bacterial additives on the quality of corn silage. J. Dairy Sci..

[38] Cai Y., Association of Self-Supply Feed Evaluation (2009). Analysis method for silage. Guidebook for Forage Evaluation.

[39] Lee S., Lee D.K. (2018). What is the proper way to apply the multiple comparison test?. Korean J. Anesthesiol..

[40] Afanador N.L., Tran T.N., Buydens L.M.C. (2013). Use of the bootstrap and permutation methods for a more robust variable importance in the projection metric for partial least squares regression. Anal. Chim. Acta.

[41] Liu Q.H., Shao T., Zhang J.G. (2013). Determination of aerobic deterioration of corn stalk silage caused by aerobic bacteria. Anim. Feed Sci. Technol..

[42] Li F., Ding Z., Ke W., Xu D., Zhang P., Bai J., Mudassar S., Muhammad I., Guo X. (2019). Ferulic acid esterase-producing lactic acid bacteria and cellulase pretreatments of corn stalk silage at two different temperatures: Ensiling characteristics, carbohydrates composition and enzymatic saccharification. Bioresour. Technol..

[43] McGarvey J.A., Franco R.B., Palumbo J.D., Hnasko R., Stanker L., Mitloehner F.M. (2013). Bacterial population dynamics during the ensiling of Medicago sativa (alfalfa) and subsequent exposure to air. J. Appl. Microbiol..

[44] Xu Z., He H., Zhang S., Kong J. (2017). Effects of inoculants Lactobacillus brevis and Lactobacillus parafarraginis on the fermentation characteristics and microbial communities of corn stover silage. Sci. Rep..

[45] Romero J.J., Zhao Y., Balseca-Paredes M.A., Tiezzi F., Gutierrez-Rodriguez E., Castillo M.S. (2017). Laboratory silo type and inoculation effects on nutritional composition, fermentation, and bacterial and fungal communities of oat silage. J. Dairy Sci..

[46] Gallo A., Bertuzzi T., Giuberti G., Moschini M., Bruschi S., Cerioli C., Masoero F. (2016). New assessment based on the use of principal factor analysis to investigate corn silage quality from nutritional traits, fermentation end products and mycotoxins. J. Sci. Food Agric..

[47] Muck R.E. (1988). Factors influencing silage quality and their implications for management. J. Dairy Sci..

[48] Kung L., Shaver R.D., Grant R.J., Schmidt R.J. (2018). Silage review: Interpretation of chemical, microbial, and organoleptic components of silages. J. Dairy Sci..

[49] Gervasi T., Pellizzeri V., Calabrese G., Di Bella G., Cicero N., Dugo G. (2018). Production of single cell protein (SCP) from food and agricultural waste by using Saccharomyces cerevisiae. Nat. Prod. Res..

[50] Yamakawa S.-i., Yamada R., Tanaka T., Ogino C., Kondo A. (2012). Repeated fermentation from raw starch using Saccharomyces cerevisiae displaying both glucoamylase and α-amylase. Enzyme Microb. Technol..

[51] Chaucheyras F., Fonty G., Gouet P., Bertin G., Salmon J.-M. (1996). Effects of a strain of Saccharomyces cerevisiae (Levucell^®^ SC), a microbial additive for ruminants, on lactate metabolism in vitro. Can. J. Microbiol..

[52] Lau M.W., Dale B.E. (2009). Cellulosic ethanol production from AFEX-treated corn stover using Saccharomyces cerevisiae 424A(LNH-ST). Proc. Natl. Acad. Sci. USA.

[53] Kung L., Robinson J.R., Ranjit N.K., Chen J.H., Golt C.M., Pesek J.D. (2000). Microbial populations, fermentation end-products, and aerobic stability of corn silage treated with ammonia or a propionic acid-based preservative. J. Dairy Sci..

[54] Filya I. (2003). The effect of Lactobacillus buchneri and Lactobacillus plantarum on the fermentation, aerobic stability, and ruminal degradability of low dry matter corn and sorghum silages. J. Dairy Sci..

[55] Arriola K.G., Kim S.C., Adesogan A.T. (2011). Effect of applying inoculants with heterolactic or homolactic and heterolactic bacteria on the fermentation and quality of corn silage. J. Dairy Sci..

